# Addressing barriers in diffuse intrinsic pontine glioma: the transformative role of lipid nanoparticulate drug delivery

**DOI:** 10.5599/admet.2214

**Published:** 2024-07-23

**Authors:** Zenab Presswala, Sheetal Acharya, Shreeraj Shah

**Affiliations:** Department of Pharmaceutical Technology, L.J Institute of Pharmacy, Lok Jagruti University, L.J Campus, Near Sarkhej-Sanand Circle, Off. S.G. Road, Ahmedabad-382 210, India

**Keywords:** Brain cancer, child cancer, nanoparticles, liposomes, nano lipid carriers, solid lipid nanocarriers, preclinical studies, clinical studies

## Abstract

**Background and purpose:**

The brainstem tumour known as diffuse intrinsic pontine glioma (DIPG), also known as pontine glioma, infiltrative brainstem glioma is uncommon and virtually always affects children. A pontine glioma develops in the brainstem's most vulnerable region (the "pons"), which regulates a number of vital processes like respiration and blood pressure. It is particularly challenging to treat due to its location and how it invades healthy brain tissue. The hunt for a solution is continually advancing thanks to advances in modern medicine, but the correct approach is still elusive. With a particular focus on brain tumours that are incurable or recur, research is ongoing to discover fresh, practical approaches to target particular areas of the brain.

**Experimental approach:**

To successfully complete this task, a thorough literature search was carried out in reputable databases like Google Scholar, PubMed, and ScienceDirect.

**Key results:**

The present article provides a comprehensive analysis of the notable advantages of lipid nanoparticles compared to alternative nanoparticle formulations. The article delves into the intricate realm of diverse lipid-based nanoparticulate delivery systems, which are used in Diffuse Intrinsic Pontine Glioma (DIPG) which thoroughly examines preclinical and clinical studies, providing a comprehensive analysis of the effectiveness and potential of lipid nanoparticles in driving therapeutic advancements for DIPG.

**Conclusion:**

There is strong clinical data to support the promising method of using lipid-based nanoparticulate drug delivery for brain cancer treatment, which shows improved outcomes.

## Introduction

**Figure 1.**
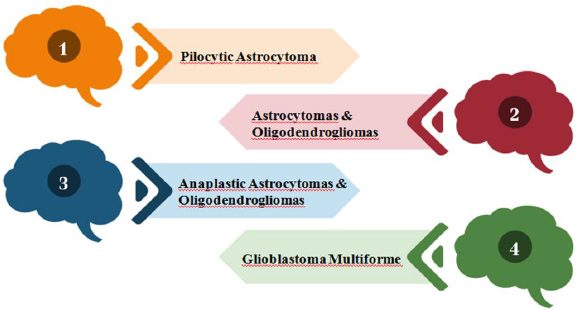
Classification of brain gliomas according to the World Health Organization

A diverse subset of gliomas called brainstem gliomas (BGs) most frequently affects young children. These tumors are known as gliomas because they develop from glial cells, a type of supporting cell in the brain. On the basis of their anatomical structure and clinical behaviour, they can be divided into groups [1,2]. Gliomas are divided into four grades by the World Health Organization (WHO) based on histopathologic characteristics like necrosis, micro vascular proliferation, cytological atypia, anaplasia and mitotic index, as shown in Figure 1 [3,4].

In 2016, the World Health Organization (WHO) implemented a significant update to the diagnostic criteria for infiltrating gliomas. This update introduced molecular markers into the classification system, including histone alterations, 1p/19q chromosome status, and isocitrate dehydrogenase mutations. Despite the remarkable progress made in the field, it is disheartening to acknowledge that the outlook for individuals diagnosed with malignant or high-grade (III and IV) gliomas remains exceedingly bleak [3,5]. Diffuse intrinsic pontine glioma (DIPG) is a pediatric malignant tumour that develops widely in the stem region of the brain, which is called pons. The usual age at diagnosis for this unsettling illness is 6-7 years old, and adults are rarely diagnosed with it. Less than 10% of patients remain alive following their delay in diagnoses for more than two years due to the location and existing treatment choices. Approximately 80% of all kinds of pediatric brain tumours that develop in the brainstem are DIPGs [6-9]. Unfortunately, DIPG has a dismal prognosis and, regardless of the type of treatment used, the median survival time is typically less than 16-24 months [1].

### History of DIPG

**Figure 2.**
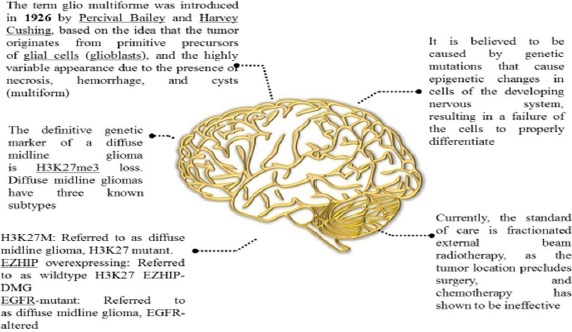
History and facts of DIPG

Since the name "glioma" was first used over 150 years ago, numerous gliomas have been identified and described [10]. Although DIPG has existed throughout human history, it wasn't until the 1920s that it was first mentioned in English medical literature. At the time, there was no neuroimaging, thus, a patient with the classic DIPG symptoms (in-turned eye, weakness on one side) was diagnosed with the disease solely by physical examination. Radiation was increasingly used to treat cancer from the 1930s through the 1950s, and it was ultimately discovered to be effective against several CNS tumours. Further, we have discussed a few DIPG facts in Figure 2 [11].

### Epidemiology and diagnosis

**Figure 3.**
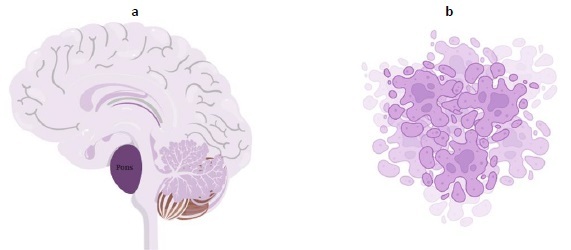
**a** - cancer arising from pons; **b** - pictorial image of cancer cells.

The tumor is aggressive, as evidenced by the fact that the majority of children survive for less than a year. Figure 3a explains cancer arising from pons and Figure 3b shows the pictorial image of cancer cells. DIPGs cannot be surgically resected due to their infiltrative nature and localization in the brainstem. In the Unites States, roughly 300-350 new cases of DIPG are identified each year [12]. Patients typically exhibit brainstem syndromes, which can occur singly or in combination and include ataxia, long tract symptoms, and cranial nerve dysfunction [1,6,13].

The preferred diagnostic method is MRI (magnetic resonance imaging), with as well as without an intravenous agent. A high-signal lesion that occupies at least two-thirds of the pons and frequently spreads laterally into the cerebellar hemispheres and peduncles as well as vertically into the midbrain and medulla [1,14]. Contrast enhancement often only accounts for 0 to 25 % of the tumour volume, making it a less noticeable feature. Cysts are small bumps found uncommon; however, necrosis, a premature cell death, can occur. Diffusion-weighted MRI often does not reveal restricted water diffusion. Advanced neuroimaging reveals that DIPGs have low cerebral blood volume and hypoperfusion, and MRI reveals a moderate rise in choline levels and a fall in acetylaspartate levels [15]. Because of the associated risk of morbidity and lack of therapeutic efficacy, a tissue biopsy is typically not necessary in cases where the MRI appearance of DIPG is so distinctive [1].

### Molecular characterization

**Figure 4.**
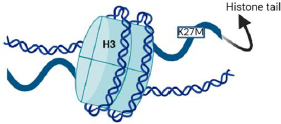
The K27M mutation in histone H3 is a characteristic feature of DIPGs.

H3K27M, MYCN, and silent are the three molecular defines groups of DIPGSs [6,16]. Nearly 80 % of DIPGs carry H3 mutations, as depicted in Figure 4, compared to around 35 % of other brain tumours [17]. H3.1 and H3.3 are the isoforms, which are expressed by the genes HIST1H3B and H3F3A, respectively, and have methionine in place of lysine as a result of the histone mutation H3K27M [18,19]. The survival, phenotype, and clinical effects of the histone mutations in H3.1 and H3.3 differ slightly from each other [20]. Histone alterations in the H3.1 subunit are frequently linked to marginally increased survival and decreased metastases. Regardless of the isoform affected, the H3K27M is generally linked to significantly inferior results compared to other H3 wild-type cases [21,22].

By inhibiting polycomb repressive complex 2 (PRC2), a gene belonging to the Polycomb group that directly and indirectly controls the DNA damage response and functions as an anticancer barrier, this mutation results in the loss of histone trimethylation and, as a result, epigenetic silencing [18,19].

DIPGs have MYC and MYCN mutations, which function as transcriptional regulators to improve gene expression throughout the genome [6,17]. Apart from this, other mutations were found, such as ACVR1, TP53, PIK3RI, and PIK3CA [6].

All relevant information about DIPG, including its ongoing treatments, new understandings, mutations, and diagnostic methods, has been compiled in Table 1.

**Table 1.** Clinical, pathological, and genetic characteristics of DIPG are summarized in the following table [1,6,23,24]

Location	Pons
Prognosis	Median Overall Survival is 8-12 Months
Age of diagnosis	6-7 years
Prevelance	10-20 % of all paderaite brain tumours, 80 % of all pediatric brainstem tumors
Clinical presentation	>50 % classic symptoms: Long tract and Cerebellar signs, Cranial nerve palsies.
	Cranial nerve VI and VII dysfunction
	Obstructive hydrocephalus
Diagnostic Tools	MRI (Common)
	Stereotactic biopsy and histological review, molecular testing.
Symptom Onset	Rapid, usually 1 month before symptoms are noticed.
Molecular testing	Next generation sequencing, DNA microarrays
Molecular subgroups	H3K27M, MYCN and Silent
Mutations	Histone 3 (H3) - 80 % - significantly worse outcomes vs H3 wild-type
	H3K27M - Isoforms H3.1 and H3.3
	ACVR1 – 30 % - co segregates with H3.1, facilitates early tumor progression
	TP53-20-40 % - often coincident with PDGFRA amplification
	PIK3RI and PIK3CA - PI3K pathway oncogenes
	MYC and MYCN aberrations - transcriptional regulators, enhance overall gene expression
Histology	Common: high-grade astrocytic, increased mitotic activity, microvascular proliferation and /or necrosis Rare: lower-grade histology, overall bland cytology.
Immunohistochemistry	GFAP, ATRX, p53, neurofilament, ki-67 immunostains
	Targeted antibodies for H3K27M, IDHIR132H
Current treatment	Only fractionated radiation at the dose of 54-59 gy or in combination of radiation therapy and chemotherapy
Treatment roadblocks	Monotherapy and combination chemotherapy- no substantial benefit
	Location- does not allow for meaningful surgical resection
	Medication delivery failure in crossing blood-brain barrier
Ongoing trials	Histone deacetylase (HDAC) and demethylase inhibitors
	Transcriptional regulators
	Immunotherapy - immune cell recruitment and/or introduction to tumour
	Drug delivery enhancement

## Study screening for formulations/ methodology

**Figure 5.**
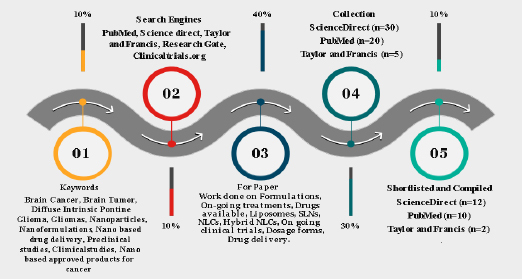
Pictorial representation of study screening in formulations available for treatment of DIPG

In order to complete this task, an extensive literature search was conducted in well-established databases such as Science Direct, ResearchGate, MEDLINE (PubMed), Scopus, Taylor & Francis, and Clinicaltrials.org. The examination of titles and abstracts of published articles, which were identified during the search, was conducted in accordance with the predetermined inclusion and exclusion criteria. Within this context, this article presents a thorough investigation and analysis of several formulations intended for the treatment of brain cancer. To fully comprehend the various therapy modalities, a thorough evaluation of the body of research, clinical trials, and scholarly articles was conducted. A variety of formulations, such as immunetherapies, targeted therapies, chemotherapeutic drugs, and combination medicines, have been included. The methods of action, efficacy profiles, possible adverse effects, and overall impact on patient outcomes have all been covered by systemic analysis. The outline of our screening using different tools is described in Figure 5.

## Treatment options

**Figure 6.**
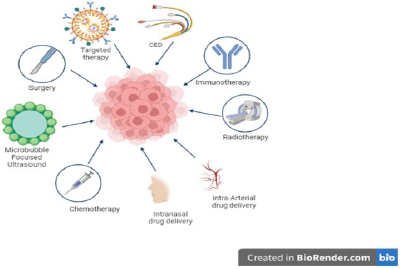
Overview of treatment options for DIPG.

Based on our literature, many treatment options are available for DIPG, as shown in Figure 6, and continuous research is ongoing to find a cure. Conventional therapeutic drugs are limited in their application due to their non-selectivity, unfavorable side effects, low efficacy, and inadequate biodistribution.

Therefore, creating delivery systems that are versatile and well-controlled is the main goal of current research efforts [25,26]. Delivering a variety of compounds to specific parts of the body through the association of therapeutic medicines with nanoparticles that have distinctive physicochemical and biological properties and engineering their paths for appropriate targeting is a promising strategy [25,27]. The utilization of carriers in protecting sensitive drugs from enzymatic degradation and the subsequent enhancement of drug bioavailability by promoting their diffusion through biological membranes is a significant area of research.

**Figure 7.**
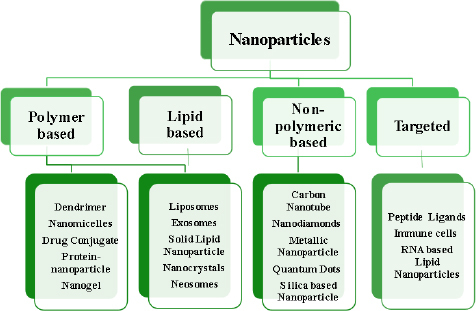
Classification of nanoparticles

A developing method for using nanotechnological systems in illness diagnosis and treatment is called nanomedicine. Nanomaterials and Nanodevices are the two primary categories into which this area of nanotechnology can be divided. Nanodevices are tiny, nanoscale devices, such as respirocytes and microarrays, capable of intelligence. Particles smaller than 100 nm in at least one dimension are found in nanomaterials [28-30]. Figure 7 describes different classes of nanoparticles.

## How nanoparticles can cross the blood-brain barrier

**Figure 8.**
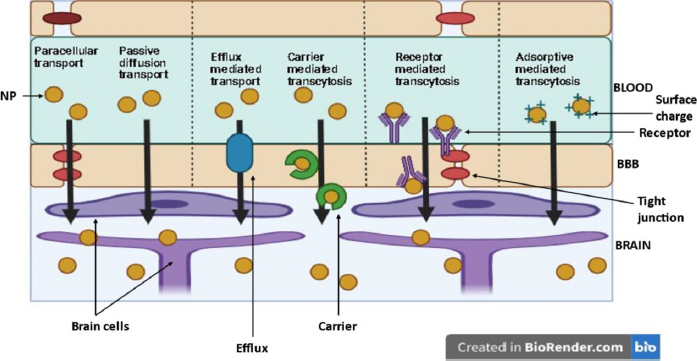
Different mechanisms of drug crossing from blood into the brain

Nanoparticles (NP) are suitable carriers for drug delivery to brain tissues as they can enhance drug permeation through the blood-brain barrier (BBB) by both active (through receptor and carrier transport) and passive diffusion (through paracellular and transcellular pathways). Figure 8 shows various pathways for NP to enter the brain [31].

### Role of size, surface charge and ligands of nanoparticles influencing BBB permeation

**Figure 9.**
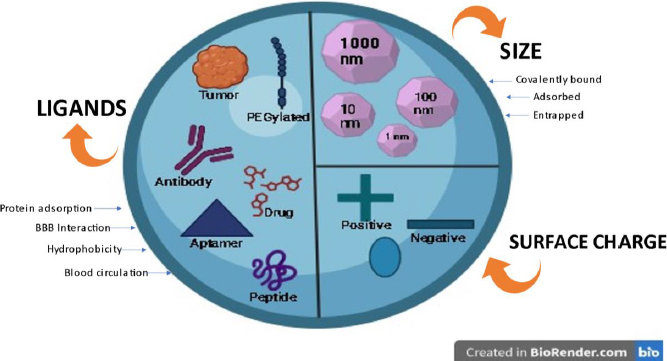
Main nanoparticle (NP) features influencing systemic delivery and blood-brain barrier (BBB) passage

Several aspects influence the effectiveness of NP systemic circulation, BBB penetration, and cellular delivery. These aspects have been outlined in Figure 9. Multiple studies have demonstrated a distinct negative link between the size of nanoparticles (NPs) and their ability to penetrate the blood-brain barrier (BBB) [32-35]. NP can be anywhere between 1 and 1000 nm in size, and they can entrap, adsorb, or covalently bind drugs to cells.

Zeta potential is a significant factor that influences the movement of nanoparticles through the blood-brain barrier (BBB). Research has demonstrated that nanoparticles (NPs) possessing a high zeta potential (indicating a strong positive charge) induce rapid toxicity at the blood-brain barrier (BBB) [32,36]. Thus, most NP formulations discussed in the literature regarding brain administration have a moderate charge ranging from -1 to -15 mV or high (between -15 and -45 mV) [37,38].

Ligand targeting is the process of adding particular ligands to the surface of NMs to enable better interaction with BBB receptors or transporters [39]. Several ligands have been attached to nanoparticles (NPs) to enhance their ability to cross the blood-brain barrier (BBB). These molecules can be classified into four distinct categories: (i) ligands that facilitate the binding of proteins from the bloodstream to specific receptors or transporters in the blood-brain barrier (BBB); (ii) ligands that directly interact with BBB receptors or transporters; (iii) ligands that enhance the electrical charge and hydrophobic properties and (iv) ligands that enhance the duration of blood circulation [32,40,41].

In the first scenario, we can incorporate poly(sorbate 80) (often referred to as Tween 80), which has the ability to adsorb apolipoprotein E and/or A-I. The surfactant facilitates the attachment of apolipoproteins, which then engage with lipoprotein receptors in the brain endothelium, allowing passage through the blood-brain barrier (BBB). In the second scenario, we have the option to incorporate multiple targeting ligands, such as those designed for the transferrin receptor (transferrin peptide, transferrin protein, or antibody against transferrin), insulin receptor, glucose transporter, and various others [40,42,43]. In the third scenario, nanoparticles (NPs) have been covered with amphiphilic peptides to enhance their absorption by blood-brain barrier (BBB) endothelial cells. Furthermore, the quantity of ligands and their receptor affinity significantly influence the transportation of nanoparticles across the blood-brain barrier (avidity). The density of ligands is determined by both the surface area of the nanoparticles and the size of the ligands. Usually, the binding strength between the ligand and its receptor decreases when the ligand is attached to nanoparticles (NPs). The avidity and selectivity of the targeted ligands are enhanced when numerous ligands are conjugated. Nevertheless, NP attraction's intensity must be adjusted to achieve efficient BBB transcytosis [44,45].

In the fourth scenario, for instance, PEGylated NPs accumulate more efficiently in the brain, which results in improved blood circulation time [45,46].

In conclusion, various factors affect the transportation of nanoparticles (NPs) via the blood-brain barrier (BBB) to varying degrees.

### Role of nanocarriers in DIPG

To boost the brain penetration of nanocarriers carrying chemotherapeutic medicines against glioma tumours, several strategies involve changing the BBB. Only those nanocarriers that can carry anticancer medications to brain tumours without disrupting the BBB are included in the subsequent discussion of nanocarriers. However, the primary justification for using nanocarriers in high-grade glioma treatment is that over 98% of medicines fail to cross the BBB [3].

### Role of nanoparticles as a diagnostic agent in DIPG

Currently, with morphological investigation of tissues or cells and with the help of imaging tools, early cancer can be diagnosed. Most imaging techniques, such as X-rays, MRIs, computed tomography (CT), endoscopies, and ultrasounds, can only detect cancer when the tissue has undergone a conspicuous change [47]. Although nanotechnology has not yet been used in clinical settings to diagnose cancer, it is presently available in a number of medical screenings and testing [48]. Nanoparticles are used to collect cancer biomarkers for cancer detection, including cancer-associated proteins, circulating tumour DNA, circulating tumour cells, and exosomes [49]. Promising nanotechnology diagnostic techniques are being created to provide on-demand, practical, and affordable cancer detection and diagnosis tools. As a result of their superior volume-to-surface ratio when compared to bulk materials, many nanotechnologies, including quantum dots, gold nanoparticles, magnetic nanoparticles, and polymer dots, are used for cancer detection. This property enables the dense coating of antibodies, small molecules, peptides, aptamers, and other substances on nanoparticle surfaces. Such compounds can bind to and identify particular cancer molecules [47].

### Role of nano formulations as a therapeutic agent in DIPG

As is common knowledge, the BBB protects against harmful substances entering the brain. Endothelial cells are arranged closely together and are found along the brain's capillaries. BBB is a filter for passive transport and is selectively permeable to water, nutrients, and hydrophobic compounds [50]. Its defense mechanism comprises the facilitated efflux of bacteria and poisons with lipid solubility via P-gp. Tight junctions are fundamental structural elements necessary for endothelial cells to function. These tight connections of endothelial cells are absent from normal capillaries of blood circulation, but the BBB endothelial cells' tight junctions shield the brain [51]. Most medications fail to pass this barrier and lose their therapeutic benefits, rendering neurological patients incurable. The most extensively researched delivery systems for BBB translocation, nanocarriers are effective in targeting or utilizing specific biological substances, receptors, carriers, or processes of the brain. Due to their promising properties and extensive uses in neurological illnesses, the class of polymeric nanoparticles and lipid nanocarriers such as liposomes, solid lipid nanoparticles, micelles, and others are receiving particular attention from neuroscientists [52]. Various invasive and non-invasive techniques focused on getting past the BBB's impediment and focusing on the necessary sick areas of the brain have been developed in recent years [53]. Hydrophilic or lipophilic neuropharmaceutical substances can be loaded into nanoscopic formulations of biodegradable/ Biocompatible/ synthetic/ natural polymers in solid or solution form, protecting them against biological and chemical risks [54]. The unique advantages of Nano formulations for neurotherapeutic drug transportation include size in the nanometer range, morphological characterization, efficient targeting by surface modification using ligands, a variety of administration routes, and stimuli-sensitive neurotherapeutic release [55].

## Role of lipid-based nanoparticles in DIPG

**Figure 10.**
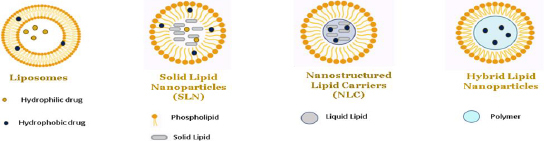
The diagram illustrates three distinct categories of lipid-based nanoparticles: i) liposomes, ii) solid lipid nanoparticles (SLNs), iii) nanostructured lipid carriers (NLCs), and iv) hybrid lipid-polymeric nanoparticles.

**Figure 11.**
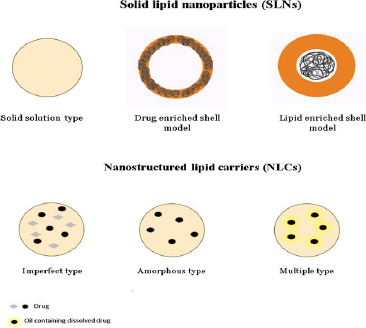
An illustrative depiction of various categories of SLNs (solid lipid nanoparticles) and NLCs (nanostructured lipid carriers). SLNs refer to solid lipid nanoparticles that can be categorized into three models: solid solution, drug-filled shell, and lipid-filled shell, where as NLCs subclasses are characterized by imperfections, types lacking a definite shape, types consisting of numerous components

In exploring the various types of nanoparticles discussed above, it is noteworthy to highlight the efficacy of lipid-based nanoparticles, such as liposomes, solid lipid nanoparticles and nano lipid carriers, in delivering hydrophilic and lipophilic drugs [56,57]. In nanoparticle-based formulations, lipids are often used to encapsulate or carry active ingredients such as phospholipids, triglycerides, phytosterols, and fatty acids. Different categories of lipid-based nanoparticles with detailed structures are shown in Figures 10 and 11.

## Role of liposomes in DIPG

Originally identified in 1965, liposomes are spherical entities comprising an internal aqueous core and an amphipathic phospholipid bilayer [58]. Liposomes possess a core-shell nanostructure that enables the loading of hydrophilic and hydrophobic compounds. Typically, pharmaceuticals that repel water are enclosed within the fatty layers of the outer shell, whereas drugs that dissolve in water are retained within the watery part of the inner core [59,60]. In the realm of liposome preparation, a multitude of techniques have been employed. These include mechanical dispersion, solvent dispersion, detergent removal, sonication, French Pressure cell (extrusion), freeze drying, micro-emulsification, membrane extrusion, and dried reconstituted vesicles [61-63].

The table presented herein offers a comprehensive summary of preclinical investigations centered around the utilization of liposomes in the context of Diffuse Intrinsic Pontine Glioma (DIPG). These studies aim to elucidate the potential therapeutic interventions and advancements in the field of DIPG research, thereby shedding light on this intriguing area of study. Preclinical studies of liposomes in DIPG have been illustrated in Table 2.

**Table 2.** Preclinical studies of Liposomes in DIPG

Drug	Formulation	Excipients	Overview	Method of Preparation	Evaluation parameters	Outcome
Docetaxel [64,65]	Gold liposomes via Intravenous route	Glutathione, transferrin, cholesterol, egg phosphatidylcho line, vitamin E	The goal of this research was to create gold-based theranostic liposomes that target the transferrin (Tf) receptor containing docetaxel (DCX) and glutathione (AuGSH). The carboxylated Vit-E TPGS (TPGS-COOH) was used as linker.	Using the solvent injection technique, a therapeutic agent (DCX) and a diagnostic agent (AuGSH) were added to the lipophilic bilayer and core of liposomes, respectively. Additionally, gold liposomes were decorated with Tf.	TEM, FTIR, NMR, Particle size, PDI In vitro cytotoxicity, Atomic force microscopy. Drug encapsulation. In vitro cellular uptake study	The results of these investigations suggest that Tf coated liposomes loaded with DC and AnGSH could be a useful platform for customised nano-theranostics. A medication loading level of around 70 % was reached for both Nano formulations.
Docotexal [66]	Liposomes via Intravenous route	Transferrin D-alphatocopheryl polyethylene glycol 1000 succinate mono-ester (TPGS), cholesterol	The creation of transferrin conjugated TPGScoated theronostic liposomes for brain cancer imaging and treatment was the goal of this study.	Solvent Injection method	Particle size, polydispersity, morphology, drug encapsulation efficiency, in-vitro release study and brain theranostic, In vivo study	
Methotrexate [67,68]	Liposomes via Intravenous route	Hydrogenated soy phosphatidylcholine (HSPC), egg-yolk phosphatidylcholine (EYPC), and 1,2-distearoylsn-glycero-3-phosphoethanolamine-conjugated polyethylene glycol MW 2000 (mPEG-DSPE), cholesterol, acetonitrile, methanol and formic acid	This study involved quantitative evaluation of the effects of liposome formulations on the in vivo release and brain delivery of methotrexate (MTX) in rats. On the basis of egg-yolk phosphatidylcholine (EYPC) or hydrogenated soy phosphatidylcholine (HSPC), two PEGylated liposomal MTX formulations were created. By measuring the released, unbound MTX in brain and plasma using microdialysis, the drug release and uptake into the brain following intravenous administration of both formulations were compared to unformulated MTX.	The ethanol injection method, which involves the pre-insertion of PEG lipid, is a notable technique in the field.	Liposome stability study in vitro, In vivo micro dialysis, Size, PDI, EE, lipid levels,	The findings presented in this study highlight the significant influence of the phospholipid used in the formulation of PEGylated liposomes on the in vivo release and brain delivery of MTX.
Irinotecan s [69,70]	Nanoliposomes via Intranasal route	1,2-dioleoyl-sn-glycero-3-phosphocholine, 1′,3′-bis[1,2dioleoyl-sn-glycero-3-phospho]-glycerol, cholesterol, and 1,2-dist earoyl-sn-glycero-3-phosphoethanolamine-N- -[methoxy(polyethylene glycol)-2000]	The effectiveness of nanoliposomal (LS) irinotecan (CPT-11) and its active metabolite, 7-ethyl-10-hydroxycamptothecin (SN-38), in DIPG patient-derived xenograft models was examined in this work.	Thin film hydration by solvent rotary evaporator. Final the solution underwent additional homogenization through the use of an ultrasonified homogenizer.	Cell viability and colony formation assays, apoptosis assay, in vitro cellular uptake and distribution, xenograft models, analysis of drug concentration in the brainstem tumor, in vivo therapy response study, immunohistochemistry, statistical analysis	
Limonene [71]	Liposomes	Trypsin-EDTA, Phosphate Buffer Saline (PBS), 3-(4,5-dimethylthiazol-2-yl)-2,5-diphenyltetrazolium bromide salt, Dimethyl sulfoxide.	This study set out to assess the effectiveness of liposomes loaded with d-limonene on U87MG cells as well as the signaling pathways connected to cell death.	Not disclosed	Not disclosed	The outcomes demonstrate that human embryonic kidney (HEK293) cells were not able to internalize the drug-loaded liposomes; instead, they could only do so in glioma (U87MG) cells.
Paclitaxel, Berberine [72]	Liposomes	Folic acid, Tween 80, Folic acid, Soybean Phospholipids, Cholesterol.	A formulation that is both intelligent and responsive to the tumor microenvironment (TME) using folic acid (FA) derivatives and mitochondria was created.PTX-Tween 80-BBR + FA-Lip, a co-modified liposome coated with Tween 80 that targets berberine (BBR) derivatives, was created.	Not disclosed	Not disclosed	Based on highly specific tumor targeting and mitochondrial targeting, the results demonstrated that PTX-Tween 80-BBR + FA-Lip can observably improve the therapeutic efficacy of chemotherapy. This can lead to new concepts and approaches for the targeted therapy of gliomas.
Octarginine [73]	Liposomes	1,2-distearoyl-sn-glycero-3-phosphocholine, Rhodamine- 1,2-dioleoyl-sn-glycero-3-phosphoethanolamine-N, PEG_2K_-DSPE.	The current study aims to create new PEGylated liposomes grafted with octaarginine (R8) peptide-HFQ lipid for targeting glioma cells.	Not disclosed	Particle size, zeta potential and polydispersity index TEM, In vitro studies.	The study's findings suggest that ligand-HFQ-lipid liposomes could potentially serve as a suitable alternative to ligand-PEG2K-lipid-modified liposomes for drug delivery in tumor targeting.
Artesunate, Temozolamide [74]	Liposomes	mPEG2k-DSPE, fluorescein isothiocyanate isomer I-dextran, Target ApoE-peptide with thiol	Adistinct mixture Artesunate-phosphatidylcholine (ARTPC) encapsulated with temozolomide (ApoE-ARTPC@TMZ) is the basis of an ApoE-functionalized liposomal nanoplatform that has been shown to effectively co-deliver dual therapeutic drugs to TMZ-resistant U251-TR GBM in vivo.	Not disclosed	Not disclosed	The preclinical promise of this innovative integrative technique to deliver combination medicines to brain tumors was demonstrated through the use of combination liposomes. By reducing the effective dosage of TMZ and thereby mitigating systemic TMZ-induced toxicity, this approach shows great potential.

## Role of solid lipid nanoparticles in DIPG

Drug-incorporated, highly ordered crystalline structure with emulsifiers characterises SLNs made up of fully crystallised lipid components. The first SLNs were created in the mid-1990s using lipids, including triglycerides, fatty acids, and waxes, with melting points greater than both body and room temperature [75,76]. SLNs offer numerous benefits, including enhanced drug protection, better nanoparticle stability, and controlled release characteristics that can be adjusted by changing the lipid composition [77]. SLNs, however, have two main issues: low drug loading capacity and poor long-term drug retention. During the storage process, a remarkable phenomenon takes place: lipids undergo a polymorphic transition from a very high-energy phase to a low-energy phase. This transition leads to the development of a meticulously well-formed crystalline structure, adding an intriguing dimension to the overall process. Additionally, it is worth noting that this transition also has an impact on the encapsulated pharmaceuticals, gradually causing them to leak out over time. Hence, the polymorphism severely restricts the amount of drug loading capability, particularly for highly pure lipids [78,79]. Various methods are used for preparing solid lipid nanoparticles, such as High shear homogenization, High-speed homogenization or ultra-sonication, Solvent emulsification/evaporation, Micro emulsions, Double emulsion, Spray drying, SLN preparation by using super critical fluid [80]. The following table highlights the preclinical studies on solid lipid nanoparticles conducted in DIPG. Preclinical studies of solid lipid nanoparticles in DIPG has been illustrated in Table 3.

**Table 3.** Preclinical studies of solid lipid nanoparticles in DIPG

Drug	Formulation	Excipients	Overview	Method of preparation	Evaluation parameters	Outcome
Carmiustine (BCNU) Temozolamide (TMZ) [81]	Solid lipid nanoparticles	Cetyl palmitate, β-hydroxybutyric acid	The objective of this study was to formulate and develop solid lipid nanoparticles (SLN) with the intention of enhancing their anti-proliferative effects against glioblastoma multiforme (GBM).	Not disclosed	size, morphology, chemical structure, zeta potential, drug encapsulation efficacy, drug release, Biocompatibility, stability were determined, and in vitro studies	The BCNU and TMZ loaded solid lipid nanoparticles (SLNs) demonstrated a noteworthy increase in their antitumor activity when compared to the free-drugs. Additionally, these SLNs were found to induce apoptosis on U87MG cells, further highlighting their potential therapeutic efficacy. Furthermore, it was observed that brain cells expressing MCT-1 exhibited a higher uptake of the specifically targeted nanoparticles.
Asiatic acid [82]	Solid lipid nanoparticles	Glyceryl tristearate, chloroform, acetic acid, L-glutamine, Poloxamer 188	The present study aimed to evaluate the effecttiveness of solid lipid nanoparticles (SLNs) loaded with AA in combating glioblastoma, a type of cancer. Additionally, the study sought to investigate the cellular uptake mechanism of these SLNs in comparison to SVG P12 cells, which are human fetal glial cells.	Solvent evaporation and Hot homogenisation technique	Size analysis and zeta potential, entrapment efficiency, HPLC, XRD, % In-vitro drug release, Cell uptake study, Apoptosis study, y fluorescence imaging and flow cell cytometry	Solid lipid nanoparticles (SLNs) may cure glioma, a brain tumor. Delivery of the drug to its target site is efficient due to these nanoparticles' drug encapsulation effectiveness. SLNs also have low toxicity to healthy cells, which is important for safe and successful therapeutic development. For glioma treatment, SLNs supplemented with ascorbic acid are promising. These SLNs are promising candidates for treating this difficult condition due to their excellent drug encapsulation efficiency and minimal toxicity.
Emodin [83]	Solid lipid nanoparticles via	Poloxamer 188, Tween 80	This study prepared and characterized solid lipid nanoparticles loaded with emodin (E-SLNs) and tested their in vitro anticancer efficacy.	High pressure homogenization (HPH)	Particle size analysis, zeta potential measurement, drug entrapment efficiency (EE), stability and in vitro drug release behavior	Results suggest lipid nanoparticle administration of EMO may be a potential cancer treatment.
Folic acid-doxorubicin (FAD) [84]	Solid lipid nanoparticles	Tween 80	The objective of this study was to establish the formulation of tween 80-coated solid lipid nanoparticles (SLNs) that would effectively encapsulate the folic acid-doxorubicin (FAD) conjugate. The ultimate goal was to achieve a targeted drug delivery system specifically designed to target brain cancer cells.	Solvent injection method	Particle size, zeta potential, surface morphology, entrapment efficiency, cytotoxicity and cellular uptake study	The results of the study have confirmed that the use of tween 80-coated solid lipid nanoparticles (SLNs) holds promise for targeted delivery of doxorubicin specifically to brain cancer cells.
Paclitaxel (PTX) [85]	Solid lipid nanoparticles	PEG 600, Poloxamer 188, lecithin, stearic acid, (1,2-dipalmitoyl-sn-glycero-3-phosphoethanolamine.	The objective of this study was to develop solid lipid nanoparticles (SLN) that have been modified with tyrosine 3 octreotide (TOC) and loaded with paclitaxel (PTX). The aim was to enable a dual-targeting approach for the treatment of tumor cells and necrosis vessels.	Self assembly	Morphology done by AFM, SEM and TEM, hydrodynamic diameter (Z average, nm), polydispersity index (PDI) and zeta potential,D rug loading and entrapment efficiency, In vitro drug release, In vitro cell culture study, In vitro tube formation study, Hemolysis test, Glioma models, Scintigraphic imaging studies, In vivo anti-tumor efficacy assay, In vivo antiangiogenic, antiproliferative, and anti-glioma activity.	The findings of this study hold significant potential for guiding the development of future DDS designs. Specifically, the utilization of diverse somatostatin analogs, including but not limited to Tyr-3-octreotate, which exhibit a heightened affinity for SSTR2, could be explored. Such an approach may pave the way for enhanced therapeutic outcomes in the field of DDS.
Zidovudine [86]	Solid lipid nanoparticles	Stearic acid, polyvinyl alcohol, aloevera gel, MTT, polyethyleneimine, trypsin.	This study develops aloe vera-modified stearic acid solid lipid nanoparticles (SLNs). Zidovudine, a common antiretroviral, was put into these SLNs. The simple and successful emulsion solvent evaporation method was used to make these SLNs. These SLNs were durable at ambient temperature and in a refrigerated environment. This suggests that SLNs could deliver drugs reliably. Overall, this study advances nanotechnology by developing SLNs utilizing stearic acid modified with aloe vera. Zidovudine's encapsulation and stability in these SLNs enable new delivery methods for this antiretroviral medication.	Emulsion solvent evaporation	The hydrodynamic size, zeta potential, TEM, entrapment efficiency and drug loading, Blood compatibility studies, Drug release, Cell culture studies, In-vitro cytotoxicity studies, In vitro cellular uptake studies.	According to the cellular absorption investigation, these nanoparticles may improve brain cell uptake of antiviral drugs and be a good drug carrier system for Glioma treatment.
Nutlin [87]	Magnetic solid lipid nanoparticles	Cetyl palmitate, ethylene glycol,	The aim of this study was to develop and evaluate solid lipid nanoparticles loaded with Nutlin for treatment of glioblastoma.	Solvent evaporation technique.	Zeta potential, SEM, TEM, encapsulation efficiency and drug loading, In-vitro release, Cell culture, Static in vitro blood-brain barrier model, BBB crossing investigation,	Nut-Mag-SLNs had great colloidal stability, stronger pro-apoptotic activity against glioblastoma cells than the free drug, and the ability to cross the blood-brain barrier. Nut-mag-SLNs are promising multifunctional nanoplatforms for glioblastoma multiforme treatment.

## Role of nano lipid carriers in DIPG

Through the substitution of fractional solid lipid components of solid lipid nanoparticles (SLNs) with liquid lipids, the formulation of nanostructured lipid carriers (NLCs) was advanced. This modification allowed for a larger drug-loading capacity within the NLCs [88]. The NLC (nanostructured lipid carrier) exhibits remarkable benefits as a drug delivery system, offering notable advancements in drug retention and enhanced drug loading capacity. However, it is worth noting that the limited solubility of medications in solid lipids poses a challenge, resulting in a comparatively low encapsulation efficiency (EE). The induction of phase separation in numerous nematic liquid crystals (NLCs) is achieved by the deliberate combination of a solid lipid with a higher oil concentration. The solubility of drugs experiences a notable increase when drug-encapsulated oily nano compartments are formed. This occurrence holds the potential to greatly enhance the drug's encapsulation efficiency [89].

**Figure 12.**
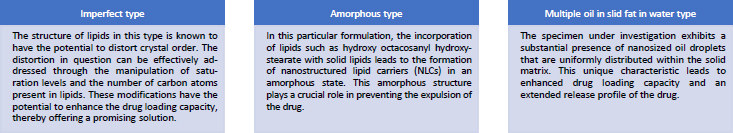
The diagram illustrates three distinct categories of nano lipid carriers.

Nano lipid carriers are basically of three types, which are named as (i) imperfect type, (ii) amorphous type and (iii) multiple oil in solid fat in water type [90]. All the three types have been explained in Figure 12

Nanolipid carriers are prepared using the same methods as solid lipid nanoparticles. According to the authors in [91], it was demonstrated that Temozolamide (TMZ) could be administered to U87MG cells more successfully through Nano lipid carriers rather than through Polymeric Nanoparticles and Solid Lipid Nanoparticles when they were created. The following table highlights the preclinical studies on nano lipid carriers conducted in DIPG. Preclinical studies of nano lipid carriers in DIPG are illustrated in Table 4.

**Table 4.** Preclinical studies of nano lipid carriers in DIPG

Drug	Formulation	Excipients	Overview	Method of preparation	Evaluation parameters	Outcome
Temozolamide Reservetol [92]	Nano lipid carriers via intranasal route	Lactoferin	The work's goal was to create NLC co-loaded with resveratrol and lactoferrin conjugated temozolomide for the treatment of glioblastoma via intranasal delivery for brain targeting.	Not disclosed	Particle size, Transmittance, Entrapment efficiency, Polydispersity index, TEM, SEM, Ex-vivo permeation studies.	According to ex-vivo experiments on permeation, at 24 hours, the percentage of resveratrol and temozolomide that permeated was 76.43 %, 88.55 % from LTR-NLC, and 25.76 and 31.10% from suspension, respecttively.Based on the results of this study, it can be said that LTR-NLC might be a useful formulation for the treatment of glioblastoma.
Temozolamide [91]	Polymeric Nanoparticles, Solid lipid nanoparticles, Nano lipid carriers.	Stearic acid, poly-(lactic-co-glycolic acid, Tween 80, polyvinyl alcohol, soyabean phosphotadylcholine, injectable soyalecithin, polyoxyl castor oil.	The delivery of Temozolomide (TMZ) has been ingeniously designed to utilize polymeric nanoparticles, solid lipid nanoparticles, and nanostructured lipid carriers. These advanced delivery systems offer promising avenues for enhancing the therapeutic efficacy of TMZ. In order to ascertain the most suitable nanocarrier for the treatment of gliomatosis cerebri, an evaluation was conducted to assess the anticancer effects of three distinct types.	T-PNPs were prepared by a solvent displacement technique, T-SLNs were prepared following the solvent displacement technique, T-NLCs were prepared by solvent diffusion method.	Particle size, zeta potential, drug encapsulation efficiency (EE), drug loading (DL) capacity, Anti-tumor efficacies.	In the realm of in vivo and in vitro experiments, it has been observed that T-NLCs have demonstrated superior performance compared to other formulations in relation to their anti-tumor activity. The NLC formulations exhibited superior glioma inhibition in comparison to both PNPs and SLNs. The findings of this study demonstrate that the delivery of TMZ to U87MG cells can be significantly enhanced through the use of NLCs, as compared to PNPs or SLNs. Moreover, this enhanced delivery method exhibits superior inhibitory efficacy. The potential utilization of T-NLCs as a promising modality for delivering chemotherapy to treat glioblastoma is worth exploring.
Docetaxel [93]	Nano lipid carriers	Propylene glycol monolaurate (Lauroglycol 90), capryolpropylene glycol monocarpylate, caprylocaproylmacrogol-8-glycerides (Labrasol^®^), polyoxyl-15-hydroxystearate (Kolliphor^®^ HS15)	The primary aim of this study was to create Nano lipid carriers of Docetaxel that possess enhanced permeation across the blood-brain barrier (BBB). This was achieved by investigating the impact of four different liquid lipids on the carrier's efficacy.	Melt emulsification method	REM, SEM, Particle size, Zeta potential, Drug entrapment study, Permeation studies using 3D In-vitro model.	In a 3D in-vitro model of BBTB/GB, NLCs showed great permeability and were able to cross the blood-brain tumor barrier (BBTB). NLCs then accumulated in glioblastoma cells. Patient-derived cells absorbed NLCs 2.4 times better than U87MG cells. The authors give a persuasive view of a prospective nanomedicine technique that they envision as simple and commercially viable. The approach has potential for further development and investigation.
Bortezomib [94]	Nano lipid carriers	Precirol ATO^®^ 5, glycerol monooleate, coumarin 6, polyoxyethylene (10) cetyl ether, MTT, hematoxylin and eosin.	For targeted distribution, nanostructured lipid carriers were loaded with two proteolytically stable D-peptides, D8 and RI-VAP (Dual NLCs).	Not disclosed	REM, SEM, Particle size, Zeta potential, Drug entrapment study, In-vivo and Ex-vivo animal imaging, In-vitro cytotoxicity, apoptosis.	Dual NLCs had the greatest therapeutic efficacy because to their improved in vitro cytotoxicity and apoptosis, longer survival rate, and excellent anti-glioma behavior in mice with intracranial gliomas. Overall, the targeting platform created for this work can deliver BTZ to glioma cells and overcome several difficulties to treat advanced brain cancer with potential therapeutic results.
SN38 (Active metabolite of Irinotecan)[95]	Nano lipid carriers	Polyvinyl alcohol, Vitamin E TPGS, MTT, Oleic acid, Cetyl palmitate.	Irinotecan's active metabolite, SN38, is 1000-fold more hazardous. However, its low solubility and stability limit its application. This study produced SN38-loaded NLCs and examined their cytotoxicity on glioma cells to overcome these factors.	Two methods were selected. Probe ultrasoniccation hot homogenization method. Modified emulsification solvent evaporation technique	Size and Morphology, Entrapment efficiency, Drug loading capacity, In vitro drug release, In vitro cell viability assay, In vitro cellular uptake, Evaluation of SN38 extraction yield from the release samples.	The average loading efficiency was 9.5 % and entrapment 81 %. The MTT test revealed that NLCs had significantly higher cytotoxicity on U87MG human glioma cells than the free drug. Thus, the study found promising NLC therapy potential for glioma.
Etoposide [96]	Nano lipid carriers	Folate, Glyceryl monostearate, Soybean phosphatidylcholine, Oleic acid, MTT, PEGDSPE	This study developed Etoposide-loaded NLCs coated with Folate for targeted delivery to FA receptor-overexpressing malignancies.	Solvent Injection tecnhique	SEM, TEM, particle size, zeta potential, polydispersity index, serum stability, in vitro drug release, tissues distribution assay, cell viability study, anti-tumor effects in vivo,	FA-ETP-NLCs were received by CT26, SGC790, and NCI-H209 cells overexpressing FA receptors. FA-ETP-NLCs also carry ETP to cancer cells, enhancing their anticancer potential. FA-ETP-NLCs may be a better tumor nanomedicine.

## Role of hybrid nano lipid carriers in DIPG

In order to accomplish several roles and to overcome the drawbacks of single-component nanomaterials, hybrid nanolipid carriers were developed as a mixed system made up of a minimum of two different categories of materials, combining the benefits of the two separate components [97]. Hybrid NLCs usually consist of a polymeric core containing a therapeutic agent enveloped by an inner lipid layer and a PEGylated outer layer [98]. Benefiting the characteristics of both lipids and polymers, hybrid NLCs were made with greater stability, sustained release and high compatibility [98-100]. Table 5 highlights the preclinical studies done on hybrid nano lipid carriers conducted in DIPG.

**Table 5.** Preclinical studies of hybrid nano lipid carriers in DIPG

Drug	Formulation	Excipients	Overview	Method of preparation	Evaluation parameters	Outcome
Paclitaxel [101]	Hydrogel hybrid system	Not disclosed	The aim of this study was to design in situ sustained-release hydrogel delivery device for glioma that combines immunoadjuvant and chemotherapeutic medication for local distribution into the resection cavity. A thermosensitive hydrogel framework (PLGA1750-PEG1500-PLGA1750) was used to embed mannitolated immunoadjuvant CpG targeting nanoparticles (MNPCpG) and glioma homing peptide modified paclitaxel targeting nanoparticles (PNPPTX&MNPCpG@Gel).	Not disclosed	Not disclosed	The study findings indicate that the utilization of a targeting nanoparticles-hydrogel hybrid system has demonstrated the ability to form a gel drug reservoir upon injection into the glioma resection cavity. This conclusion is supported by in vitro as well as in vivo results. The potential targeting of residual infiltration glioma cells by the sustained-release PNPPTX, leading to the production of tumor antigens, is an intriguing avenue of investigation. Therefore, this gel has the potential to augment the therapeutic efficacy for glioma.
Docetaxel [102]	Lipid polymer hybrid nanoparticles	PLGA, soybean lecithin, caumarin-6, arginine-glycine-aspartic (RGD)	The primary aim of this study was to develop a targeted delivery system for the anticancer drug docetaxel. To achieve this, a novel approach involving the synthesis of hybrid nanoparticles composed of lipids and the biodegradable polymer poly (D,L-lactide-co-glycolide) (PLGA) was employed.	Solvent extraction evaporation method	Size, zeta potential, encapsulation efficiency, *in vitro* drug release, *in vitro* cellular uptake, tumor spheroid penetration, growth inhibition of tumor spheroid, cytotoxicity of docetaxel, *in vivo* anti GBM effects, *in vivo* imaging	Upon administration of docetaxel-loaded arginineglycine-aspartic acid lipid nanoparticles (RGD-L-P), a noteworthy observation was made regarding the median survival time of rats afflicted with glioblastoma multiforme (GBM). The recorded median survival time was documented to be 57 days. The results demonstrate a notable fold increase of 1.43, 1.78, 3.35, and 3.56 in comparison to rats treated with L-P (*P* < 0.05), PLGA-P (*P* < 0.05), Taxotere (*P* < 0.01), and saline (*P* < 0.01), respectively.

Apart from brain delivery, lipid-based nanoparticles are widely used in oral delivery, pulmonary delivery, topical delivery, and ocular delivery.

## Toxicity of lipid-based nanoparticles

Lipid-based nanoparticles have several benefits compared to other types of nanoparticles. Lipid nanocarriers such as liposomes, SLNs, and NLCs are ideal for incorporating both hydrophilic and lipophilic drugs, one of the major advantages of a combination of drugs that give dual targeting. They also have other advantages in terms of biocompatibility, shielding bioactive compounds from chemical deterioration, site-specific controlled drug delivery, and high drug loading capacity, these nanocarriers are superior to the others in many ways. Stability during sterilization and ease of scaling up are two more benefits. At last, there is no reported toxicity for SLNs and NLCs up to date. Physiological lipids, generally accepted as safe (GRAS) excipients, make up the majority of these nanocarriers [75]. Before getting marketing authorization for these nanocarriers, their toxicity profile must be studied [103]. Further, we discuss the role of lipid-based nanoparticles in cytotoxicity, genotoxicity and hepatotoxicity.

### Cytotoxicity

The biocompatibility of all lipid-based nanoparticles is tested by determining their cell viability or cytotoxicity. A cytotoxicity assay is conducted to determine the potency of the anticancer molecule. Cell viability is tested as evidence in cytotoxicity assay [75]. In lipid-based nanoparticles, different cell lines tolerate the lipids used at high dose levels [104]. It is reported that most of the cell lines tolerate 1 mg/ml lipid SLNs/LNCs [105]. Additionally, surfactants are crucial to the stabilization of SLNs. Cell viability is unaffected by the use of 1 mg/ml of the cationic surfactant cetyltrimethylammonium bromide (CTAB). However, some studies have indicated that cationic surfactants impact the cell membrane's integrity [106].

### Genotoxicity

The most important model in this case is the induction of DNA damage because exemplary genotoxins can cause genetic alterations without causing cell death, which can initiate carcinogenesis. However, there are many studies reporting that lipid-based nanoparticles produce no genotoxicity. One study reported that No DNA damage was observed in A549 cells by the negatively charged SLNs [107,108]. However, the drug and drug-loaded SLNs reduced cell viability below 50% at the concentration chosen for genotoxicity testing [109].

### Hepatotoxicity

When a formulation enters the bloodstream, it is very important to check whether the RBC cells are damaged and to ensure this hemolysis assay is performed, which determines the damage of RBC that a foreign material causes [75]. A study revealed that even at a dose of 1 mg/ml, the SLNs made of polysorbate 80 and glycerol monostearate exhibit minimal hemolysis [110].

## Nano-based drug approved for cancer therapy by regulatory authorities.

The first drug delivery systems to be launched in the market for cancer treatment were liposomes and are still most widely used due to their several positive points such as biocompatibility, biodegradability and non-immunogenicity [111,112]. The approved nano-based formulations for brain cancer treatment are listed in Table 6.

**Table 6.** Approved nano-based products for brain cancer

Product^TM^	Drug	Dosage form	Company	Approval year
Doxil [113]	Doxorubiicn	Liposome	Ortho Biotech	1995 (FDA)
Caelyx [114]	Doxorubicin	Liposome	Schering-Plough	1996 (EMA)
DaunoXome [115]	Daunorubicin	Liposome	Galen	1996 (FDA)
Myocet [114]	Doxorubicin	Liposome	Teva UK	2000 (EMA)
Mepact [116]	Mifamurtide	Liposome	Millenium	2009 (EMA)
Ameluz [117]	5-aminolevulinic acid	Nanoemulsion	Biofrontra GmbH	2011 (EMA)
Marquibo [118]	Vincristine	Liposome	Spectrum	2012 (FDA)
NanoTherm	Iron oxide/Ferric oxide	Metallic Nanoparticles	Magforce	2013 (EMA)
Onivyde [119]	Irinotecan	Liposome	Merrimack	2015(FDA)
Vyxeos	Daunorubicin, Cytarabine	Liposome	Jazz Pharmaceuticals	2018 (EMA)

## Clinical trials for DIPG

**Figure 13.**
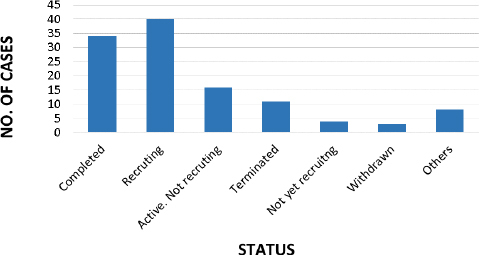
Graphical representation of clinical trials going on for DIPG

As per clinicaltrials.org (Last accessed on 20/10/2023, there are 116 studies conducted for DIPG, out of which only 34 no. of studies are completed up to date and 40 studies are found to be recruiting, presented in Figure 13 [120].

Table 7, provided below, presents a comprehensive overview of the studies that have been completed since 2020.

**Table 7.** Clinical studies completed in DIPG

Description	Drug	Formulation	ClinicalTrials.gov identifier
In this phase I/II trial, patients with a recently diagnosed diffuse intrinsic pontine glioma are treated, and the side effects of the panobinostat nanoparticle formulation MTX110 (MTX110) are investigated. Through the inhibition of certain enzymes required for cell growth, the panobinostat nanoparticle formulation MTX110 may be able to stop the proliferation of tumor cells.	Panobinostat Convection enhanced delivery	Nanoparticles	NCT03566199
In children with diffuse intrinsic pontine glioma who have finished focused radiation therapy, this is a Phase I and Early Efficacy Study using Convection Enhanced Delivery (CED) of irinotecan liposome injection (nal-IRI) Using Real Time Imaging with Gadolinium.	Irinotecan Convection enhanced delivery	Liposomes	NCT03086616

Table 8 presents a comprehensive overview of ongoing studies that have been actively recruiting participants since the year 2020.

**Table 8.** On going clinical studies completed in DIPG

Description	Drug	ClinicalTrials.gov identifier
In children recently diagnosed with diffuse intrinsic pontine glioma (DIPG), the purpose of this prospective, open-label, single-arm, multicenter clinical research is to evaluate the safety and therapeutic efficacy of combining Nimotuzumab with concurrent radiochemotherapy. The objective response rate serves as the main outcome, while the 1-year overall survival rate is a seconddary observation. The study tackles the necessity of assessing the combination therapy's possible advantages and safety in treating juvenile DIPG patients.	Nimotuzumab+ CRT (concurrent IMRT and TMZ)	NCT04532229
This work investigates gemcitabine, which has been shown to effectively penetrate the blood-brain barrier and inhibit DIPG cell lines. Gemcitabine presence in DIPG tissue after systemic treatment is the main aim, whereas intratumoral gemcitabine concentrations are the secondary objective. Before undergoing routine surgery, participants receive a single IV dose that makes it easier to analyze biopsy samples and measure gemcitabine penetration. The purpose of this research is to overcome the difficulties associated with treating this aggressive pediatric brain tumor by gaining understanding into gemcitabine's effectiveness against DIPG.	Gemcitabine	NCT02992015
The goal of this study is to determine whether treating DIPG patients according to the aforementioned methodology and stratifying them based on their MR perfusion score improves their survival outcomes. In addition to the standard MRI performed upon diagnosis, newly diagnosed DIPG patients will have an MRI perfusion scan, which will allow them to be categorized as having hyperperfused or hypoperfused tumors. Weekly low-dose Bevacizumab treatments will be administered to the hyperperfused individuals along with traditional routine radiation therapy. Patients with hypoperfusion will be administered ultra-low-dose radiation fractionation, which is the same as a biological dosage of standard RT.	Drug: bevacizumab injection radiation: ultra-low-dose RT	NCT04250064
In children with progressing diffuse midline gliomas (DMG), this study intends to evaluate the safety and viability of employing focused ultrasound with microbubbles and neuro-navigator-controlled sonication to temporarily break the blood-brain barrier (BBB). Through the use of this non-invasive method, the BBB can be opened at particular points surrounding the tumor, improving the way oral etoposide is delivered. The prognosis for diffuse midline gliomas is poor, especially for Diffuse Intrinsic Pontine Gliomas (DIPG). This study investigates a unique strategy to maximize systemic adverse effects while optimizing drug delivery and treatment success. The BBB's successful opening and shutting will be confirmed by routine MRIs.	Etoposide; oral, 50 Mg, focused ultrasound therapy	NCT05762419
In this pilot trial, children with recently diagnosed high-grade gliomas (HGG) with TRK fusion are evaluated for disease control and survival rates. After two cycles of larotrectinib monotherapy, patients receive maintenance medication for responders. Patients who show a steady course of illness or a partial response receive combination therapy or focused radiotherapy. Pre-surgery effects of larotrectinib are investigated in a surgical cohort. In addition to evaluating the safety of larotrectinib monotherapy, combination therapy, and radiation therapy, the trial intends to enroll 15 patients for disease control and safety assessment.	Larotrectinib	NCT04655404
In children and adolescents with glioblastoma, diffuse intrinsic pontine glioma (DIPG), anaplastic astrocytoma, and gliomatosis cerebri, the HIT-HGG-2013 study investigates the possibility of valproic acid, a histone deacetylase inhibitor, augmenting the effects of radiotherapy and chemotherapy (temozolomide). It attempts to compare the results with the HIT-HGG-2007 study in order to assess the safety and therapeutic efficacy of valproic acid in treating these aggressive brain tumors. Originally, an autophagy inhibitor (chloroquine) was included in the trial, but it was later canceled since Resochin junior was not available.	Temozolomide + valproic acid	NCT03243461
In this phase I trial, patients with primary central nervous system (CNS) cancers that have returned (recurrent) or are resistant to treatment (refractory) are being treated for the adverse effects and optimal dose of volitinib. By inhibiting some of the enzymes required for cell growth, volitinib may be able to stop the proliferation of tumor cells.	Savolitinib	NCT03598244
For pediatric patients (ages 3 to 21) with progressing brain cancer or newly diagnosed diffuse intrinsic pontine glioma (DIPG), indoximod-based chemo-radio-immunotherapy is being investigated in the GCC1949 project, an open-label phase 2 trial funded by the NCI. When paired with conventional therapy, inhibiting the IDO pathway—which is essential for immune regulation—is thought to improve antitumor immune responses and may lead to better results. Up to 140 patients may be included in the Johnson and Munn-led study, which is categorized according to the patients' receipt of radiation therapy and focuses on glioblastoma, medulloblastoma, and ependymoma. iRANO standards will evaluate results.	Indoximod, temozolamide, cyclophosmphamide, etoposide, lomustine	NCT04049669
In this phase I/II trial, mebendazole is being investigated in conjunction with conventional treatments for juvenile gliomas. Mebendazole is prescribed with vincristine, carboplatin, and temozolomide for patients with low-grade gliomas, and with bevacizumab and irinotecan for patients with high-grade/diffuse intrinsic pontine gliomas. Gross complete resection without significant neurologic deficit is the goal of surgical resection. Doses of metronidazole increase from 50 to 200 mg/kg per day. A "3+3" design is used to monitor safety. The most tolerable dose is established, effectiveness is assessed, and patients proceed with maintenance treatment while having their overall survival and lack of progression observed by magnetic resonance imaging (MRI).	Mebendazole vincristine carboplatin temozolomide bevacizumab irinotecan	NCT01837862

## Conclusion

Patients with DIPG still have a bad prognosis. Contrary to other malignant gliomas for which active chemotherapeutic medicines have been identified, the only established treatment for this illness is chemotherapy administered in conjunction with radiation. As a result of the clinical manifestations, molecular characterization, formulation improvements, and drug delivery system discussions in this article, it can be concluded that the use of nanoparticulate drug delivery systems is a possible tactics for the treatment of DIPG. The lipid-based nanoparticulate drug delivery system for brain cancer demonstrates remarkable efficacy owing to its unique capability to traverse the blood-brain barrier, thereby facilitating precise and targeted drug delivery. The utilization of nanoparticles presents a promising avenue for achieving controlled release kinetics, thereby mitigating potential side effects and augmenting the therapeutic efficacy of various treatments. The superiority of these agents is underscored by their exceptional biocompatibility and safety profile, as well as their remarkable specificity for targeting cancer cells. The promising strategy of utilizing lipid-based nanoparticulate drug delivery for brain cancer treatment is supported by robust clinical evidence, demonstrating improved outcomes. The scalability and cost-effectiveness of this approach further strengthen its potential in the field.
